# Annotations

**Published:** 1891-08-01

**Authors:** 


### Annotations.

**A Hopeful Departure.**

There is a "Society of Hypnology" in Paris, and its annual meetings, extending over several days, have just been held. The striking thing about the society is that although it is formed to discuss questions which, in a sense, are purely medical, yet its members include physicians, many magistrates, lawyers, and men of science. Hypnotism, or Hypnology, is just one of those things which requires the very best intellect and conscience which the world possesses for its elucidation; and not for its elucidation only, but also for the safe-guarding of hypnotised persons, and of those who practice the hypnotic art. It is said that nearly ninety per cent. of people in France are capable of being hypnotised; it seems also that many more persons than has hitherto been suspected can act as hypnotisers. It is very necessary, therefore, that the lines of legitimate practice should be as speedily as possible laid down. Another subject which would be advantaged by the collaboration of medical and non-medical minds is that of lunacy and lunatics. Here it is plain that lawyers and men of general science, but more especially of mental science, can greatly help the purely medical judgment. No one, and particularly no medical man, would wish to undervalue the medical intellect. But it is obvious to every real student of human nature that the human mind is a thinking organ which is subject to many limitations. The most powerful individual intellect in the world cannot throw the same mental candle-power on a particular object as many intellects can throw. For it is manifest that between intellects of the first and second order there is so little difference that it is often impossible to say what exactly the difference is, and wherein it consists. We do not, therefore, think less of the medical intellect when we urge that there are many medical or partly-medical subjects on which laymen, such as lawyers, scientists, politicians, and intelligent men of the world, could throw much additional light, and bring us a great deal nearer the practical truth and justice of things. Medical men are very jealous of outsiders. This jealousy is a survival from a not very scientific past, and it is quite unworthy of the true, laborious, and philosophical medical scientists of the present time. The medical practitioners of the last and some previous centuries could not afford to take the intelligent public frankly into their confidence. Their actual knowledge was of the most meagre kind, whilst their traditions and practices were often contemptible. To have sought the criticisms of outsiders would have been to court their own destruction. But all that is long since past and gone. There are no more truly scientific workers than the medical scientists of the present time. What remains to be done is for them to admit that medical science, like all other science, is for the world, and not for the comparative handful of persons who at any particular moment constitute the medical profession.

**Sir Charles Russell and the Mad Doctors.**

The verdict in favour of Mrs. Cathcart is a distinct victory for Sir Charles Russell over the medical men who had decided that the lady was insane. But, although Mrs. Cathcart has been pronounced sane by a jury and has been set at liberty, it does not follow that the lunacy doctors came to a wrong conclusion on the evidence which was brought before them. In other words, whilst it has been declared that Mrs. Cathcart is now sane, it has not been proved that she was not insane at the time when the lunacy doctors gave their certificates. All men who are scientific and human at the same time must be thankful that the case has been tried. It would be well if many other similar cases could be tried in open court, and with the advantage of skilled counsel on both sides. It should not be sufficient in any case of alleged lunacy merely to prove that a man departs from a popularly understood standard of sanity. Even if, in any particular instance, it can be shown that a person fails to reach an ill-defined standard of sanity, it does not, therefore, follow that that person should be deprived of his liberty. In all lunacy investigations there should not be one main question asked but two—first, Is the alleged lunatic really insane? and, secondly, Is his insanity of such a kind as to make it imperative that he shall be deprived of his liberty? These two points are essentially distinct, though they are ordinarily looked upon as identical. All that the law requires in order that a particular individual may be deprived of his liberty is a certificate of lunacy. But it is clear that a further certificate than one of mere lunacy ought to be demanded, namely, a certificate which distinctly affirms that the alleged lunatic is dangerous, either to himself or to other people. It seems to us remarkable that, notwithstanding the enormous number of lunacy cases that have to be dealt with every year, the law should still be in such a chaotic, uncertain, and unsatisfactory condition as it is. But it is not merely the law that is in an unsatisfactory state; the opinion and the practice even of medical specialists is just as chaotic and illogical. It is not creditable to lunacy specialists that the facts should be as they are. We want a standard of sanity defined. We want, moreover, lunacy divided into kinds and degrees. It is necessary also that it should be declared when and in what circumstances an alleged lunatic ought to be entirely deprived of his liberty, when partly deprived, and when he may safely be left to carry out his own devices. Everybody knows very well that there are hundreds of insane people going about, that is, people who are below the recognised standard of sanity. Many of these men and women are actually earning their own living. They are insane, but they are not dangerously insane. Compared with a sober-minded man, an ill-tempered child, or a flighty and hysterical woman, or even a jibbing horse, may be said to be insane. A medical man cannot but feel a certain amount of shame in that lunacy, which has been left by the law in the special care of the medical profession, is still a kind of *terra incognita* for all sorts of litigation, chicanery, and trickery to unscrupulous persons, and of indefinite misery to the parties immediately concerned. It is quite certain that the subject, though difficult to deal with, could be, and ought to be, mapped out with more clearness and precision than has ever yet been attempted. Doctors give themselves too much to the physics of the thing. The pathology, the material changes of the brain, are what they devote their attention to. That is well and right so far as it goes. But the practical world, the law, the lunatics themselves and their friends, require the external facts to be dealt with. From a practical point of view, these are a million times more important than the mere alteration of brain structure. When the world asks for practical guidance, and the doctors by way of answer proceed to describe pathological changes, they do nothing but give a stone when they are asked for bread. The world insists, common-sense insists, moral duty insists, that medical men shall deal with lunacy and with every other disease in such a practical and rational way as shall ensure the greatest measure of safety and happiness to the suffering person, and also the greatest measure of safety and happiness to all those who are in any way connected with him.

**The Oxford Infirmary: Heathen or Christian?**

The managers of the Radcliffe Infirmary, Oxford, have decided to take immediate steps for the improvement of their hospital. This, after their somewhat energetic protest that the infirmary was so faultless as not to need any improvement, is a prompt and honourable repentance. The improvement is to take the form of an entirely new male ward, and of the re-arranging of the old block for administrative purposes. The cost of the alterations will be ten thousand pounds. On the principle that half a loaf is better than no bread, we hail this outlay with satisfaction. But how much better would it have been for the sick poor, how much more honourable to medicine, and how much more worthy of our most ancient, most splendid, and most venerated University, if a noble building, with all the newest aids of science, and all the advantages of outward beauty and distinction, could have been added to the colleges and halls of Oxford! It is plain that, even yet, Oxford fails to realise the dignity and tenderness of medicine. In this respect she is still heathen rather than Christian. A new ward, and some re-arrangement of old structures, may meet the bare demands of antiseptic medicine and surgery, and may save the patients from being poisoned at the place whither they have gone in search of health. But for Oxford, the rich, the beautiful the world-famous, to lodge its books and its learning amid all the sumptuous surroundings which wealth and art can supply, and to put its sick poor and its academic medicine to the indignity of bare walls and the cheapest utility—this is a practical commentary on the beauty of charity which we never expected our greatest University to put forward. It is not yet too late for more generous and more dignified counsels to prevail.

